# Results of the Global Burden of Disease study for schizophrenia: trends from 1990 to 2021 and projections to 2050

**DOI:** 10.3389/fpsyt.2025.1629032

**Published:** 2025-09-05

**Authors:** Zhonghua Zhan, Jiaming Wang, Tongping Shen

**Affiliations:** School of Information Engineering, Anhui University of Chinese Medicine, Hefei, Anhui, China

**Keywords:** Global Burden of Disease, schizophrenia, APC, predictions, SDI

## Abstract

**Introduction:**

Schizophrenia represents a substantial aspect of the overall burden associated with mental disorders and ranks as the third leading cause of disability worldwide.

**Methods:**

Utilizing data from the Global Burden of Disease (GBD), Injury, and Risk Factors Study, this research examines global trends in the prevalence, incidence, and disability-adjusted life years (DALY) attributable to schizophrenia, with stratification by age, sex, and socio-demographic index (SDI).

**Results:**

Between 1990 and 2021, the prevalence of schizophrenia increased from 13.62 million to 23.18million, the incidence rose from 883,000 to 1.223 million, and the age-standardized disability rate (ASDR) escalated from 8.76 million to 14.82 million, reflecting increases of over 70.1%, 38.5%, and 69.2%, respectively. However, global age-standardized estimates have remained relatively stable. The male-to-female burden ratio for schizophrenia in the general population has shown consistency over the past three decades, with men exhibiting a higher risk compared to women. As regional life expectancy continues to improve, an increase in the burden of schizophrenia is anticipated.

**Discussion:**

Notably, the burden is more pronounced in economically stable, high-income countries within high SDI regions, while it is exacerbated in low SDI regions due to inadequate health policies. Projections indicate that the prevalence, incidence, and overall burden of schizophrenia will continue to rise through 2050. Consequently, policymakers across all nations should revise existing policies and implement measures, including the establishment of comprehensive systems for early diagnosis and efforts to mitigate the stigma associated with schizophrenia, to proactively address the anticipated increase in the disease’s burden.

## 1 Introduction

Schizophrenia is characterized as a cognitive and behavioral disorder that impacts early brain development, presenting a range of psychotic symptoms such as hallucinations, delusions, and disorganized behavior and speech (1). Schizophrenia is a severe and often lifelong mental illness, with its onset typically occurring in late adolescence or early adulthood (2). Notably, suicidal behavior represents the predominant cause of mortality in the initial phases of the disorder (3).

Individuals diagnosed with schizophrenia necessitate prolonged therapeutic intervention and often exhibit an impaired capacity for self-care during acute episodes. Schizophrenia has a wide range of impacts on respiratory, cardiovascular, sexual, neurological, and skin health (4), imposing a heavy economic burden on society and families (5).

Compared to women, men are at a higher risk of suffering from schizophrenia, about 1.4 times higher than women (6). Although the disorder has been known for a long time, a precise definition and etiology are still lacking (7). People with schizophrenia have a 10 to 25 years shorter life expectancy compared to the general population (8). Much of this mortality stems from unhealthy lifestyles combined with low income and limited medical care, and physical and psychological changes and increased comorbidities may also contribute to the decrease in life expectancy (9).

In addition to the significant social and employment challenges linked to the disorder, which can lead to unemployment rates as high as 90%, individuals diagnosed with schizophrenia experience enduring cognitive deficits (10). As a condition that carries considerable stigma, schizophrenia is frequently neglected by those affected, particularly in developing nations. Moreover, a decline in a country’s socioeconomic status is correlated with an increase in the stigmatization of mental health disorders, potentially resulting in an underappreciation of the impact of schizophrenia in nations with lower socioeconomic standing.

The GBD 2021 study provides globally representative and comparable estimates of schizophrenia for the current state of the global epidemiology of schizophrenia. This study aims to systematically characterize the global epidemiology and burden of disease of schizophrenia using the GBD 2021 database, to predict future trends, and to reveal differences between regions and countries through geographic stratification and temporal trend analyses, and thus to propose targeted prevention strategies.

The objectives of this study were as follows: (1) to characterize the disease burden of schizophrenia and its spatial and temporal trends by sex and age from 1990 to 2021; (2) to explore the associations with schizophrenia at the country level on a global scale and whether such associations are affected by the level of SDI. The findings of this research enhance the overall comprehension of the epidemiological characteristics and trends associated with schizophrenia, thereby assisting government policymakers in modifying current policies and implementing measures aimed at mitigating the future burden of this mental health disorder.

## 2 Methodology

### 2.1 Data sources

The Global Burden of Disease (GBD) study, conducted by the Institute for Health Metrics and Evaluation (IHME), assesses the impact of disease and injury across more than 200 countries and territories. Despite the prevalence of schizophrenia as a significant psychiatric disorder, its burden has not been comprehensively quantified in all global regions. We collected data on the incidence, prevalence, and disability-adjusted life years associated with disease and injury from 1990 to 2021. The SDI is a composite measure that incorporates the total fertility rate, the average educational attainment of individuals aged 15 years and older, and the per capita income ranking. The SDI indicates a nation’s overall social development, with values ranging from 0 to 1, and is classified into categories of low, low-middle, medium, medium-high, and high. Furthermore, the world is segmented into 21 regions based on distinct geographic characteristics, including East Asia, South Asia, and Western Europe.

The Human Development Index (HDI) encompasses macro-level indicators of human development, which illustrate the extent and accessibility of health resources. The data about the HDI is sourced from the United Nations Development Programme (UNDP, http://hdr.undp.org/en/data). The Age-Sex Ratio (ASR) is recognized as a dependable metric adjusted for age distribution, thereby mitigating the effects of variations in age structure and population size.

### 2.2 APC modeling

The principal aim of our research was to examine the temporal trend in prevalence from 1990 to 2021 by determining the annual percentage change in prevalence utilizing the “age-period-cohort” (APC) model (11) as a metric for time trends. To ensure the validity of the secondary modeling, the Estimated Annual Percentage Change (EAPC) was employed to bolster the robustness of the findings. The secondary objective was to evaluate the independent effects of age, period, and cohort (12).

### 2.3 Statistical analysis

In this study, we assessed trends in the burden of schizophrenia by employing the EAPC to quantify variations in age-standardized rates, specifically the age-standardized incidence rate (ASIR), age-standardized prevalence rate (ASPR), and age-standardized disability-adjusted life years (DALY) rate. We conducted a Pearson correlation analysis to investigate the relationship between EAPC and the Human Development Index (HDI) for the year 2021.

To project the global burden of schizophrenia through the year 2050, we initially calculated age-specific incidence rates for distinct five-year age cohorts (ranging from 0–4 years to over 95 years) from 1990 to 2021. We then employed connected-point regression models to identify years exhibiting significant shifts in age-standardized rate (ASR) trends across different age groups. Temporal trends from 1990 to 2021 were analyzed by fitting a connected-point model, allowing for the specification of up to six connecting points within the analytical framework.

Subsequently, we multiplied the predicted age-specific incidence rates by the projected global population figures for each age group, as provided by the United Nations Population Division (https://population.un.org/wpp/downloads?folder=Standard%20Projections&group=Population), to estimate the total number of cases. Finally, we computed the direct age-standardized rate by dividing the overall number of cases by the total population within each age group. A p-value threshold of less than 0.05 was established to denote statistical significance, and all statistical tests were conducted as two-sided analyses.

### 2.4 Data visualization

We used packages including ggmap, ggplot2, tidyr, reshape and RcolorBrewer for data visualization. The dplyr package was used for data cleaning. The ggmap was used to visualize the schizophrenia EAPC for 204 countries and regions.

All statistical analyses and visualizations were performed utilizing R software (version 4.3.0). This study complied with the ethical guidelines for protecting human subjects outlined in the Declaration of Helsinki. It posed no risks or potential harm to participants and did not involve commercial interests, thereby qualifying for exemption from institutional review board approval.

## 3 Results

### 3.1 The burden of schizophrenia at global and regional levels

The summary analysis of global schizophrenia incidence and prevalence data from 1990 to 2021 reveals significant changes in total volume and stratified heterogeneity in its epidemiological characteristics, as follows:

(1) Overall trends in incidence and prevalence.

Incident cases: The global number of new schizophrenia cases increased from 883,493 in 1990 (95% UI: 721,489–1,065,740) to 1,223,221 in 2021 (95% UI: 1,008,219–1,473,083), showing a significant increase in absolute numbers. However, the age-standardized incidence rate (ASIR) showed an overall downward trend, with an annual decline of 0.04% (EAPC=−0.04; 95% CI: -0.04 to -0.03).

ASDR: It increased from 8,762,312 cases in 1990 (95% UI: 6,477,261–11,263,750) to 14,816,611 cases in 2021 (95% UI: 10,926,460–19,095,362), with the global ASDR showing an upward trend (EAPC=0.04; 95% CI: 0.03 to 0.05).

(2) Trend differences by SDI stratification.

ASIR: The High SDI and High-middle SDI groups showed an upward trend (EAPC=0.14 for High SDI, 95% CI: 0.12 to 0.15); the Middle SDI, Low-middle SDI, and Low SDI groups showed a downward trend, with the Middle SDI group having the most significant decline (EAPC=-0.11, 95% CI: -0.12 to -0.1).

ASDR: Only the High SDI group showed a downward trend (EAPC=-0.01, 95% CI: -0.05 to 0.03); the other four groups all showed an upward trend, with the High-middle SDI group having the most significant increase (EAPC=0.24, 95% CI: 0.22 to 0.26).

(3) Geographical regional differences.

ASIR: The region with the highest number of new cases in 2021 was South Asia (314,673 cases); 7 regions showed an upward trend, with Eastern Europe having the most obvious increase (EAPC=0.23, 95% CI: 0.18 to 0.28); 11 regions showed a downward trend, with High-income North America having the most significant decline (EAPC=-0.09, 95% CI: -0.15 to -0.04).

**Figure 1:**
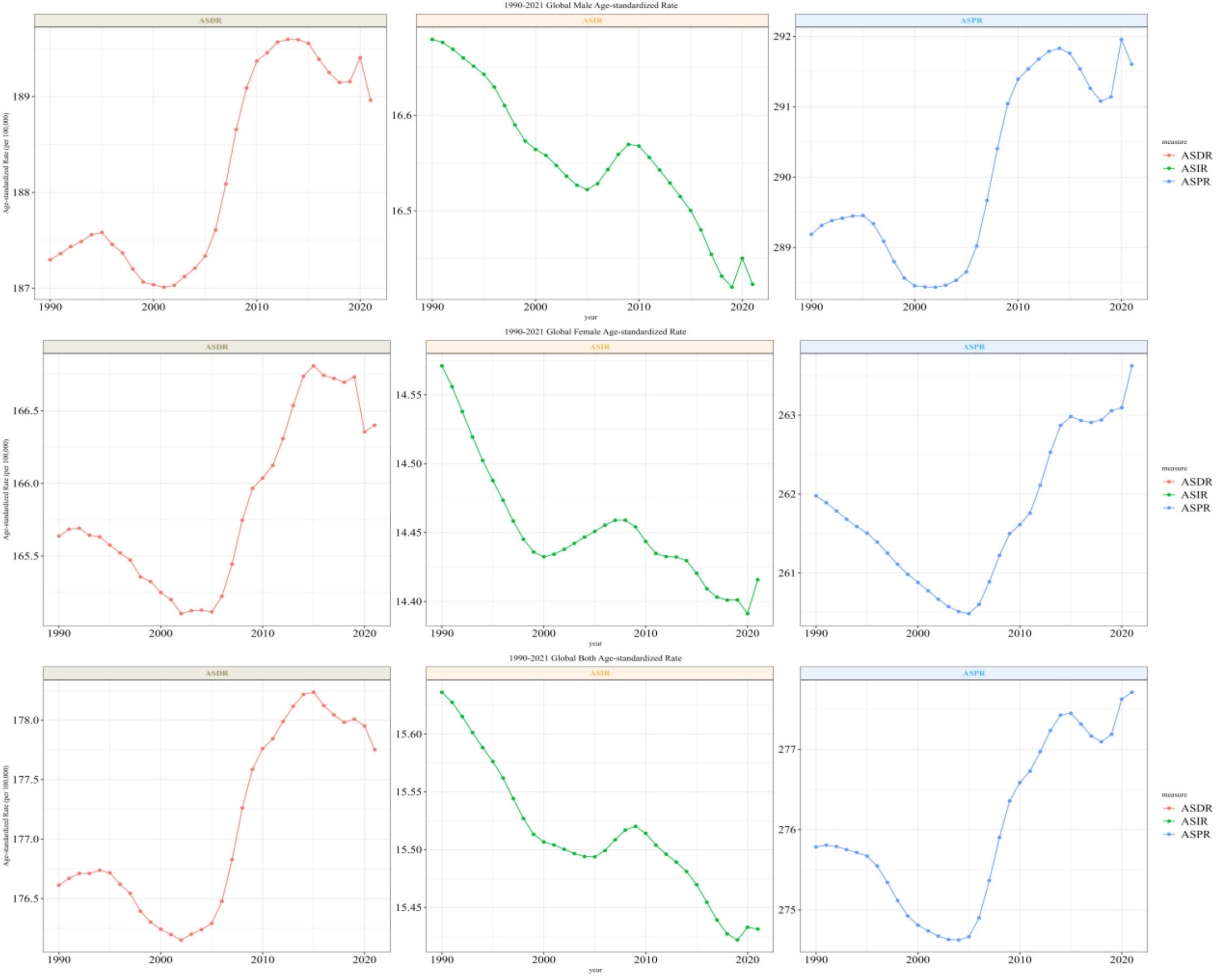
Trends in the overall global burden of schizophrenia, 1990–2021.

**Figure 2:**
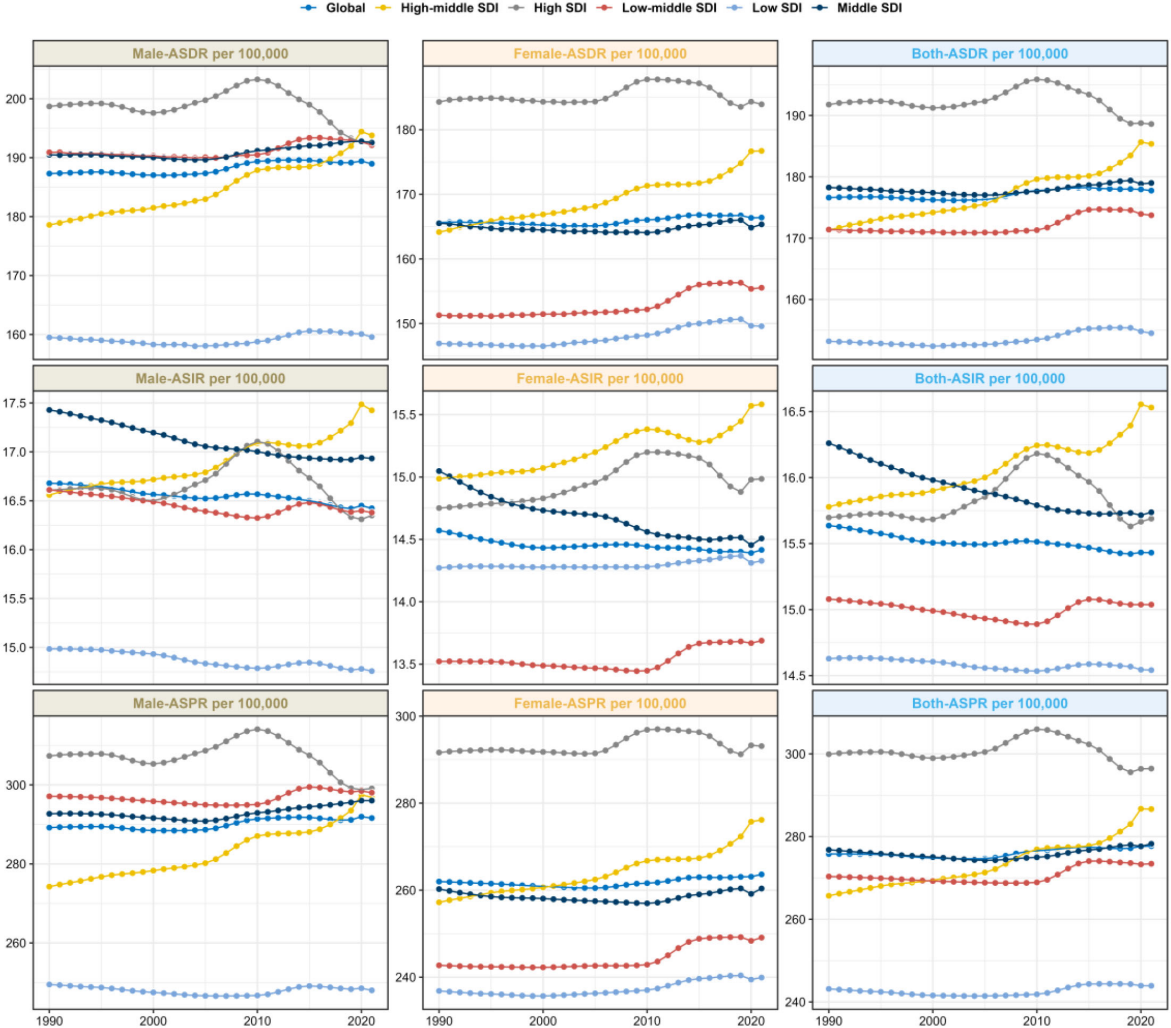
Trends in the burden of schizophrenia, globally and in the five SDI regions, 1990–2021.

**Figure 3:**
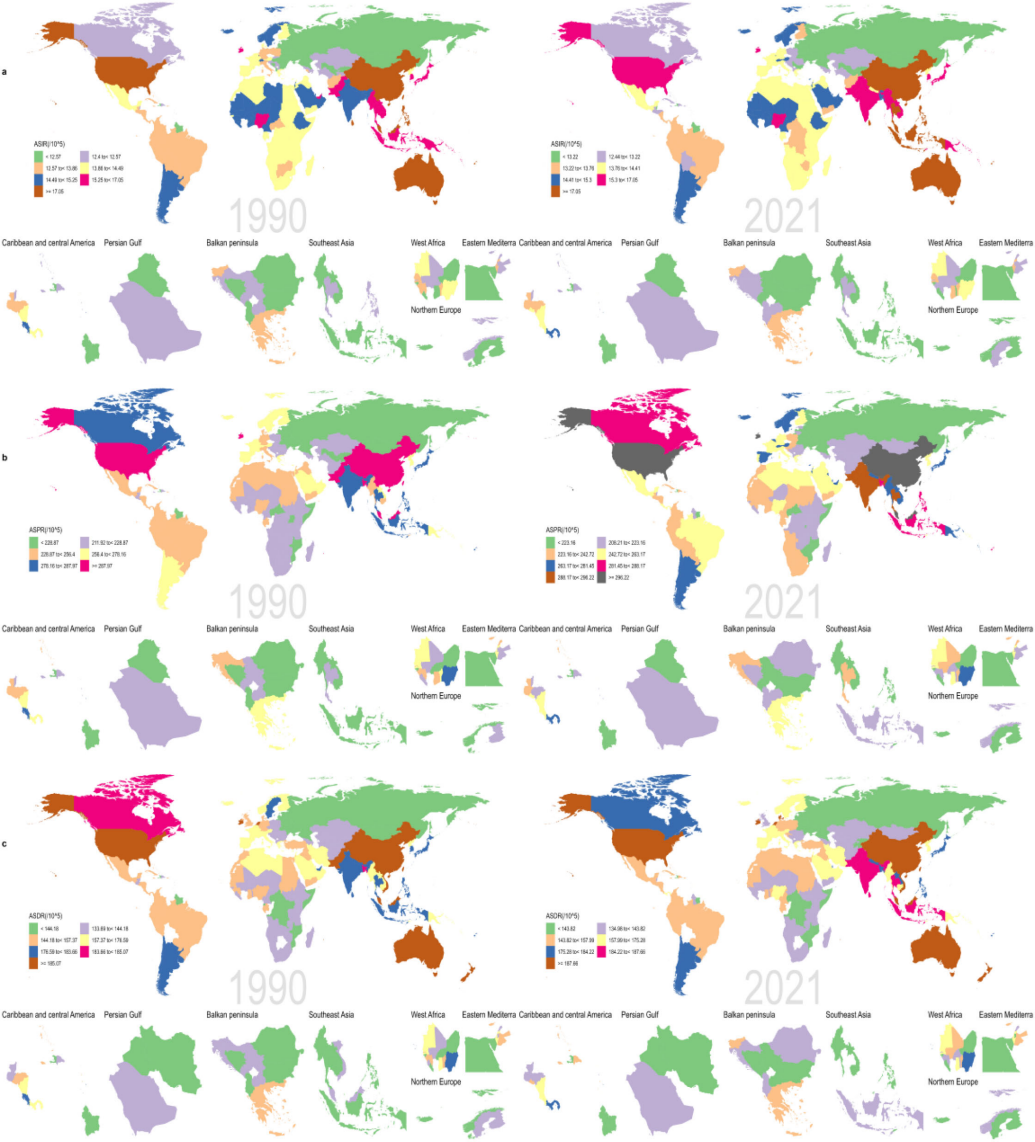
Map showing **(a)** age-standardized incidence, **(b)** age-standardized prevalence, and **(c)** age-standardized DALY for schizophrenia for 204 countries and territories between 1990 and 2021.

ASDR: 6 regions showed a downward trend, with High-income North America having the most obvious decline (EAPC=-0.12, 95% CI: -0.18 to -0.07); 15 regions showed an upward trend, with Eastern Europe having the most significant increase (EAPC=0.28, 95% CI: 0.22 to 0.35) (see **Table 1**, **Figures 1**–**3**).

**Table 1:** Global incidence of schizophrenia and EAPC, 1990–2021.

	1990		2021		1990-2021
	Incident cases	ASIR per 100,000	Incident cases	ASIR per 100,000	EAPC
	No. *10^2^ (95% UI)	No. (95% UI)	No. *10^2^ (95% UI)	No.(95% UI)	No. (95% CI)
Overall	8834.93 [7214.89-10657.4]	15.64 [13.04-18.62]	12232.21 [10082.19-14730.83]	15.43 [12.74-18.62]	-0.04 [-0.04 to -0.03]
Sex					
Female	4077.3 [3322.45-4931]	14.57 [12.13-17.41]	5638.73 [4636.88-6809.81]	14.42 [11.85-17.4]	-0.03 [-0.03 to -0.02]
Male	4757.64 [3886.57-5721.48]	16.68 [13.92-19.83]	6593.48 [5445.31-7914.79]	16.42 [13.6-19.75]	-0.04 [-0.05 to -0.04]
SDI					
High SDI	1438.38 [1189.91-1719.7]	15.7 [13.01-18.8]	1524.9 [1267.14-1831.8]	15.69 [12.95-18.76]	0.04 [0 to 0.08]
High-middle SDI	1840.7 [1564.55-2157.13]	15.78 [13.53-18.4]	2015.11 [1725.51-2367.57]	16.53 [14.15-19.33]	0.14 [0.12 to 0.15]
Middle SDI	3111.92 [2537.81-3750.53]	16.26 [13.65-19.43]	3910.71 [3214.94-4713.93]	15.74 [13.01-18.96]	-0.11 [-0.12 to -0.1]
Low-middle SDI	1758.4 [1401.26-2174.37]	15.08 [12.27-18.34]	3135.93 [2518.94-3862.96]	15.04 [12.21-18.29]	-0.01 [-0.02 to 0.01]
Low SDI	678.56 [529.73-844.42]	14.63 [11.83-17.88]	1636.92 [1275.45-2036.59]	14.54 [11.76-17.87]	-0.02 [-0.03 to -0.01]
Region					
Andean Latin America	52.49 [39.64-68.02]	13.35 [10.25-17.01]	95.05 [73.02-121.4]	13.36 [10.31-16.96]	0.01 [0 to 0.02]
Australasia	42.54 [37.56-47.97]	20.09 [17.77-22.63]	55.51 [48.49-63.05]	20.19 [17.87-22.74]	0.02 [0.01 to 0.02]
Caribbean	47.2 [35.97-60.69]	12.41 [9.67-15.64]	59.92 [46.51-75.78]	12.36 [9.55-15.61]	-0.02 [-0.04 to -0.01]
Central Asia	89.6 [67.52-115.89]	12.44 [9.61-15.88]	122.22 [93.56-155.4]	12.43 [9.55-15.74]	0 [-0.01 to 0]
Central Europe	154.23 [122.59-192.6]	12.48 [9.82-15.62]	125.4 [100.78-155.05]	12.6 [9.97-15.82]	0.03 [0.02 to 0.03]
Central Latin America	236.45 [183.76-297.35]	13.66 [10.92-16.91]	365.21 [291.9-451.25]	13.61 [10.88-16.81]	-0.02 [-0.03 to -0.01]
Central Sub-Saharan Africa	70.9 [53.42-91.06]	13.88 [10.77-17.56]	184.69 [141.2-236.88]	13.79 [10.85-17.57]	-0.02 [-0.03 to -0.01]
East Asia	2595.99 [2214.25-3007.3]	18.16 [15.66-20.98]	2448.85 [2090.48-2862.92]	18.32 [15.77-21.17]	-0.04 [-0.07 to -0.01]
Eastern Europe	252.47 [206.72-306.96]	11.18 [9.18-13.55]	212.45 [175.19-256.96]	11.73 [9.65-14.21]	0.23 [0.18 to 0.28]
Eastern Sub-Saharan Africa	247.24 [190.99-309.91]	14.29 [11.56-17.68]	608.47 [472.25-761.43]	14.16 [11.46-17.53]	-0.02 [-0.03 to -0.02]
High-income Asia Pacific	275.05 [224.11-333.76]	15.04 [12.24-18.31]	228.04 [187.12-277.23]	15.14 [12.25-18.55]	0.14 [0.06 to 0.22]
High-income North America	506.2 [419.23-609.31]	17.41 [14.42-20.77]	548.17 [459.15-651.91]	16.68 [13.81-19.82]	-0.09 [-0.15 to -0.04]
North Africa and Middle East	488.36 [372.86-615.72]	14.21 [11.23-17.66]	930.42 [735.53-1164.56]	14.07 [11.05-17.66]	-0.04 [-0.04 to -0.03]
Oceania	11.43 [8.63-14.63]	17.04 [13.34-21.36]	24.73 [19.12-31.84]	17.05 [13.41-21.67]	0 [-0.02 to 0.01]
South Asia	1700.21 [1369.97-2088.1]	15.2 [12.42-18.42]	3146.73 [2538.49-3824.52]	15.26 [12.45-18.51]	0.02 [0 to 0.03]
Southeast Asia	863.21 [680.48-1060.67]	17.23 [14.03-21.06]	1279.97 [1046.74-1559.99]	17.34 [14.18-21.06]	0.07 [0.05 to 0.09]
Southern Latin America	76.09 [57.1-96.93]	15.23 [11.56-19.33]	105.85 [81.1-135.26]	15.25 [11.64-19.37]	-0.01 [-0.02 to 0]
Southern Sub-Saharan Africa	74.94 [59.79-91.8]	13.94 [11.41-16.96]	120.4 [97.62-146.02]	13.83 [11.29-16.73]	-0.01 [-0.02 to -0.01]
Tropical Latin America	226.3 [182.9-275.07]	13.75 [11.31-16.61]	325.47 [269.14-394.19]	13.75 [11.35-16.6]	0 [-0.01 to 0.01]
Western Europe	557.79 [466.52-667.98]	14.21 [11.84-16.95]	537.02 [450.55-645.11]	14.08 [11.57-16.97]	-0.02 [-0.04 to -0.01]
Western Sub-Saharan Africa	266.27 [209.51-331.17]	14.97 [12.19-18.43]	707.61 [555.13-878.2]	14.95 [12.13-18.41]	-0.01 [-0.02 to -0.01]

(4) National-level heterogeneity.

ASIR: In 2021, the country with the highest number of new cases was India (243,679 cases), and the lowest was Niue (0 cases); 103 countries showed a downward trend, with the most significant declines in the United Kingdom (EAPC=-0.25), Finland (-0.19), and Nepal (-0.13); 72 countries showed an upward trend, with the most obvious increases in Denmark (1.85), Trinidad and Tobago (0.59), and the Russian Federation (0.35) (see S1 for details).

ASDR: In 2021, the country with the highest ASDR was China (3,445,845 cases), and the lowest was Tokelau (2 cases); 61 countries showed a downward trend, with the United Kingdom having the most significant decline (EAPC=-0.34, 95% CI: -0.48 to -0.19); 132 countries showed an upward trend, with the Kingdom of Denmark having the most obvious increase (EAPC=1.77, 95% CI: 1.44 to 2.11) (see S2 for details).

### 3.2 Connection point regression analysis

**Figure 4:**
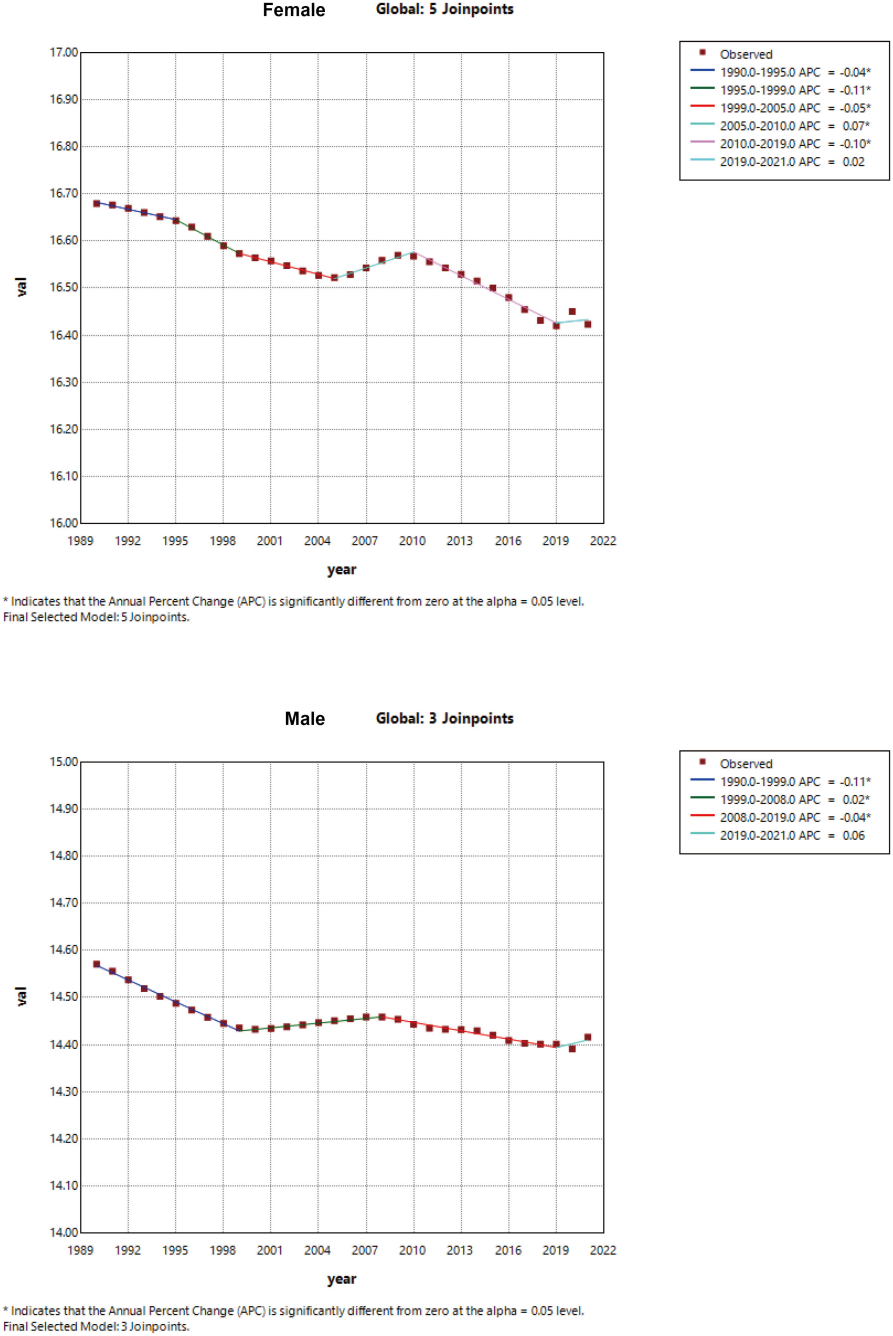
Joinpoint regression analysis of the global incidence of schizophrenia in men and women, 1990-2021.

To dissect the stage-specific characteristics and turning points of schizophrenia incidence, this study conducted Joinpoint regression analysis on the age-standardized incidence rate (ASIR) of global schizophrenia from 1990 to 2021, see **Figure 4**. A long-term continuous decline was observed, with an Annual Average Percent Change (AAPC) of −0.0352 (95% CI: −0.0396 to −0.0308) in females and −0.0484 (95% CI: −0.057 to −0.0398) in males. However, between 2019 and 2021, disrupted by the COVID-19 pandemic, the incidence rates of females and males rebounded slightly, with an Annual Percent Change (APC) of 0.06 and 0.02, respectively. In breakpoint identification, the female data were optimally fitted with 3 breakpoints (e.g., an APC of −0.11 during 1990–1999, indicating a significant decline), while the male data were optimally fitted with 5 breakpoints (e.g., an APC of −0.07 during 2005–2010, suggesting an accelerated decline). All breakpoints were validated as genuine via permutation tests (P < 0.05), and the breakpoint around 2019 coincided with the onset of the pandemic, highlighting gender-based heterogeneity.

### 3.3 Age and gender specific burden of schizophrenia

**Figure 5:**
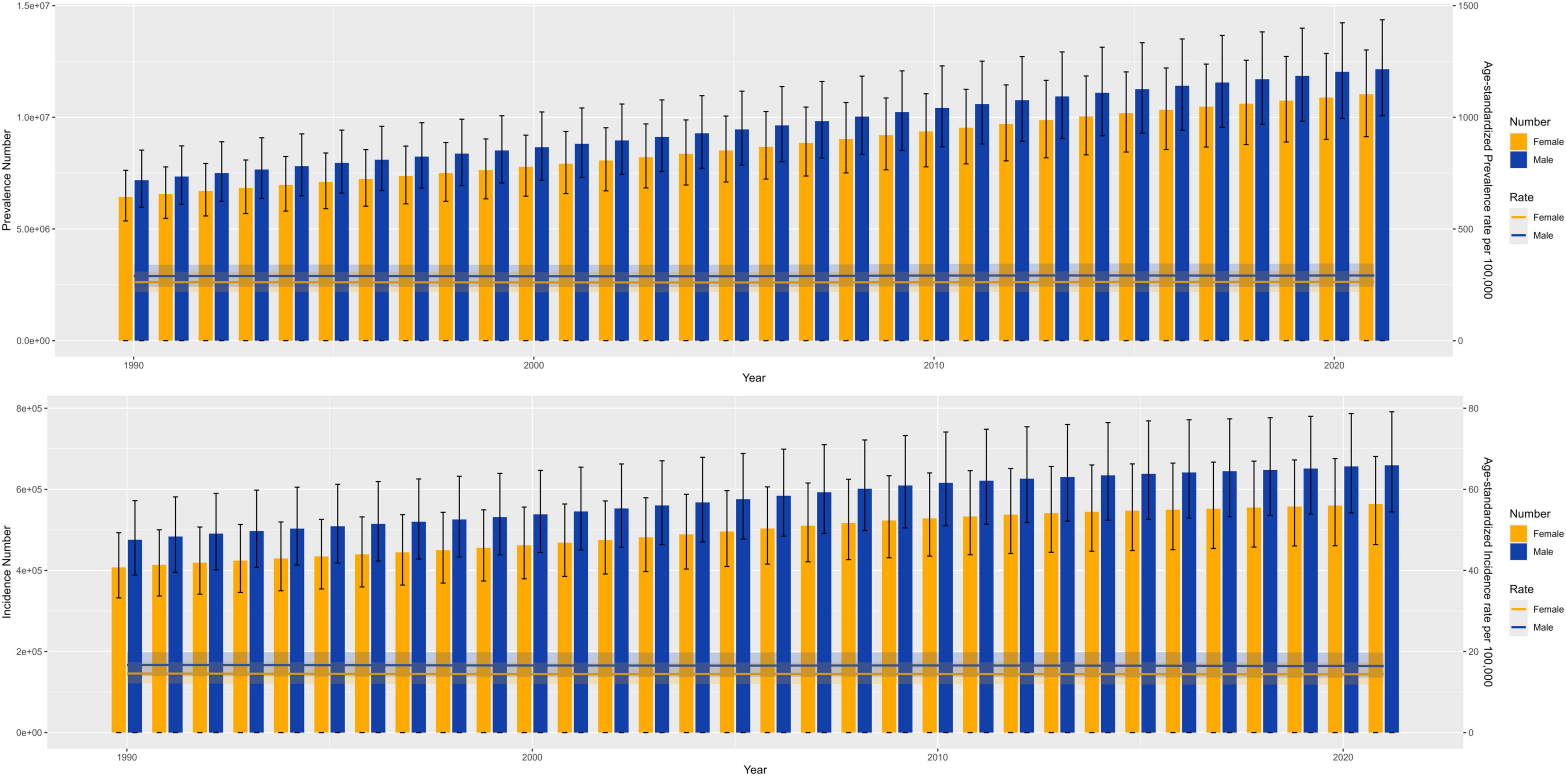
Global trends in the number of males and females with schizophrenia.

**Figure 6:**
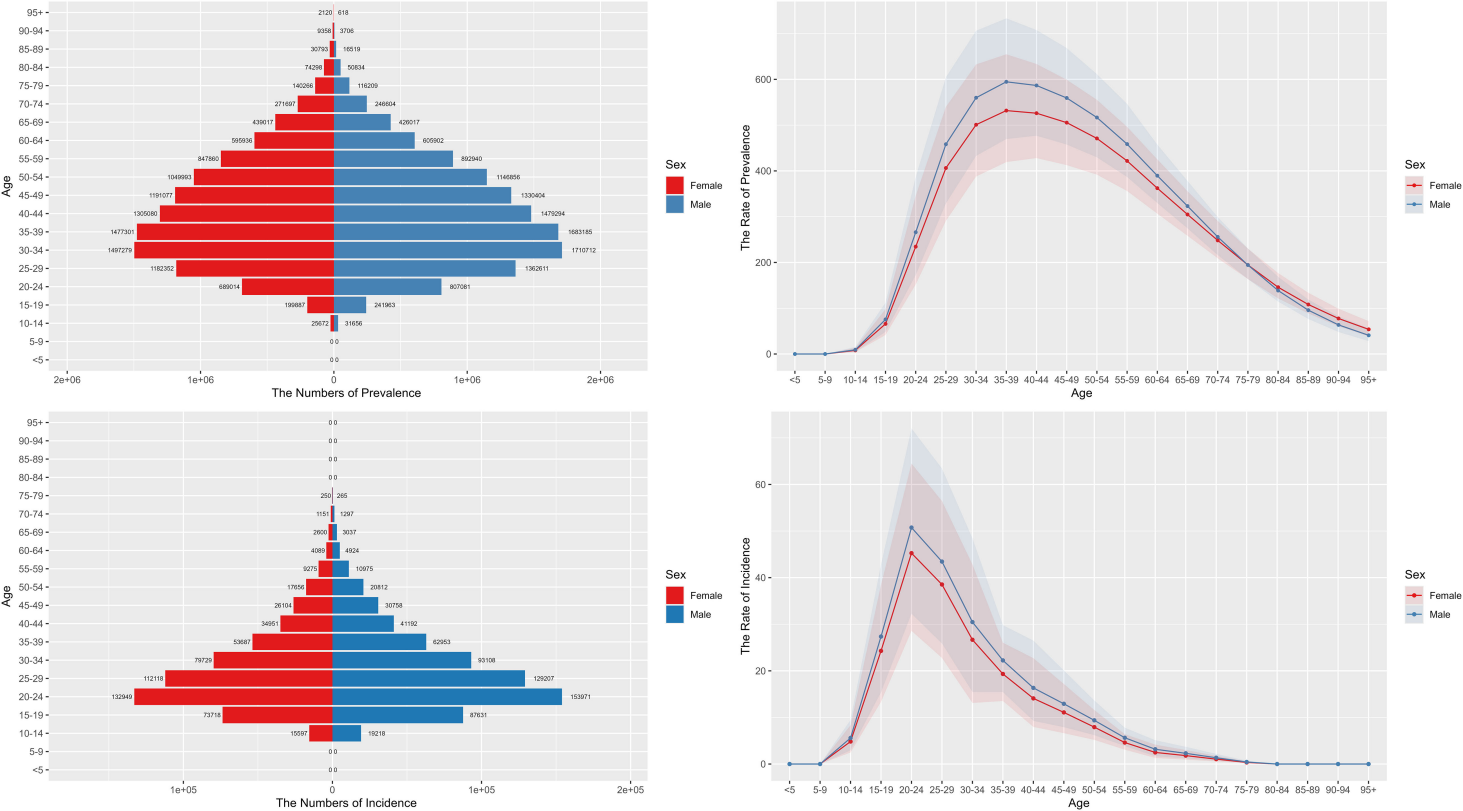
Global age distribution of males and females with schizophrenia.

From 1990-2021, there is an increasing trend in the incidence and prevalence of schizophrenia in both males and females globally, and both incidence and prevalence are higher in males than in females, see **Figure 5**. The prevalence of schizophrenia in males and females peaks in the 35–39 age group and then shows a decreasing trend. The incidence in males and females is a little earlier, with a significant rise in the 10-14-year-old group, peaking in the 20-24-year-old group, and then gradually declining in the subsequent age groups, see **Figure 6**.

### 3.4 Changes in EAPC by gender across the 22 SDI regions

**Figure 7:**
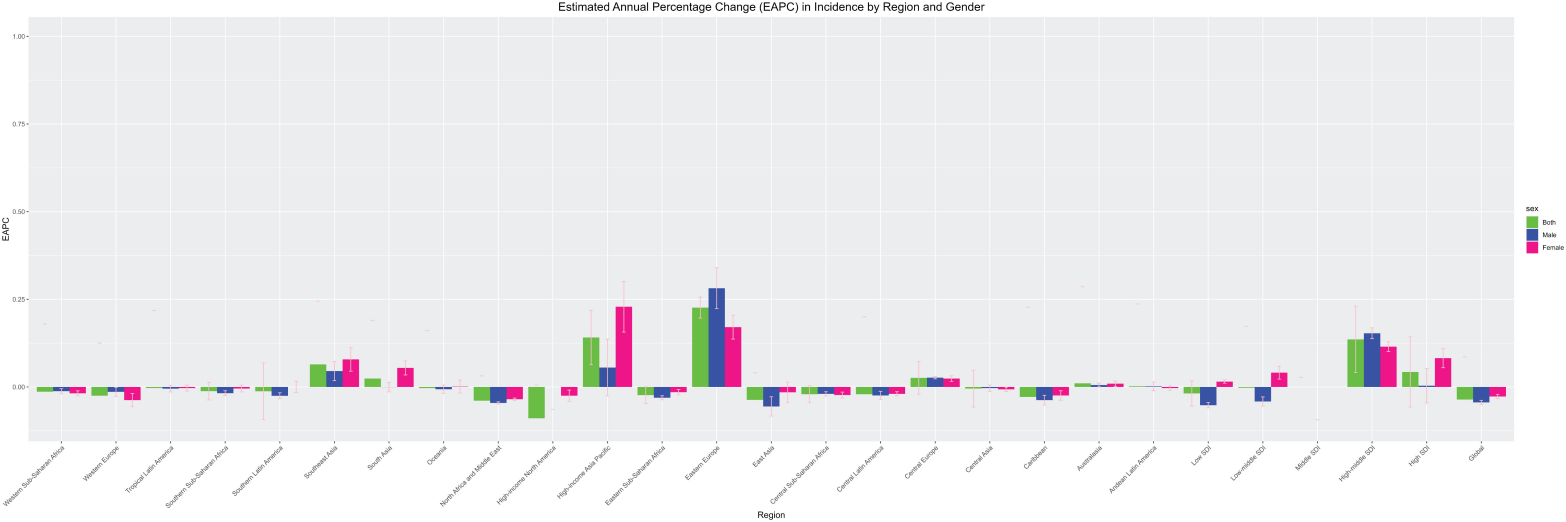
Changes in EAPC by gender in 22 SDI districts.

Looking at the various SDI regions, the increasing trend of schizophrenia in males and females showed a different status, with the burden of females being overall higher than that of males. Also the manifestation of schizophrenia varies across SDI regions. In regions such as Southeast Asia, High-income Asia Pacific and High SDI, the increasing trend of females is higher than that of males, and in regions such as Eastern Europe and High-middle SDI, the increasing trend of males is higher than that of females, see **Figure 7**.

### 3.5 Correlation between HDI and EAPC

**Figure 8:**
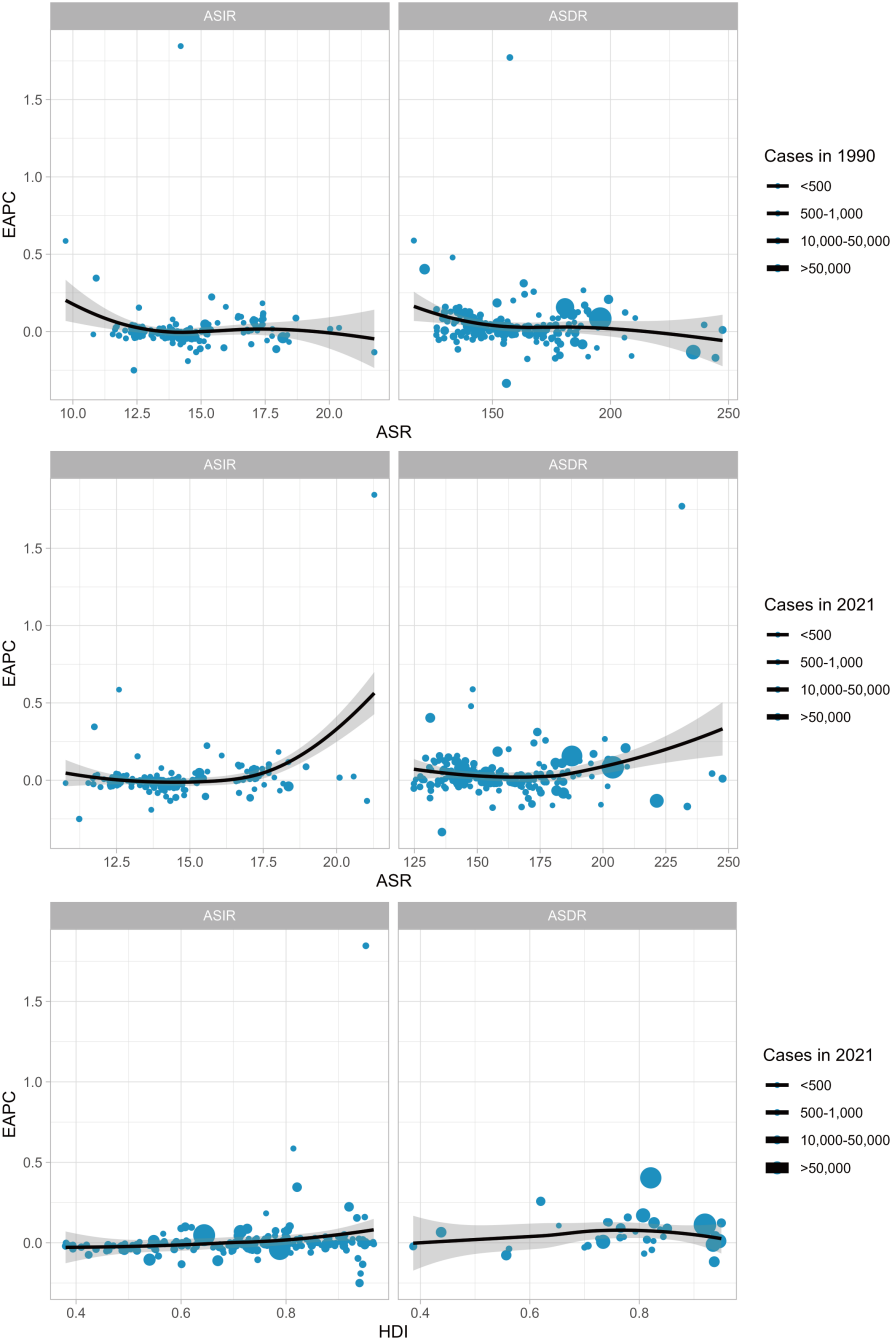
Correlations were calculated using Pearson correlation analysis.

There was no significant relationship between EAPC and incidence for 1990 incidence (cor=-0.041, p=0.56), nor was there a significant relationship for ASDR (cor=-0.149, p=0.033). There was a positive correlation between EAPC ASR for 2021 incidence (cor=0.26, p < 0.001), and no significant ASDR relationship (cor=0.14, p=0.04). We assessed the relationship between HDI (2021) and found no significant relationship between EAPC and ASIR and ASDR (ASIR: cor=0.17, p=0.017; ASDR: cor=0.07, p=0.66), see **Figure 8**.

### 3.6 Decomposition analysis

**Figure 9:**
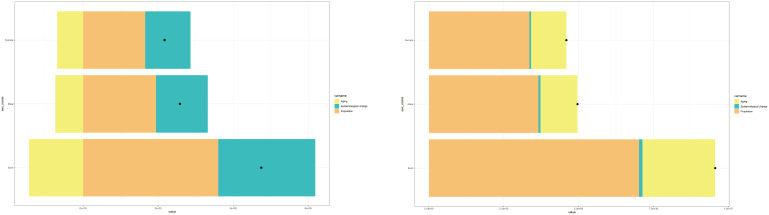
Decomposition analysis, 1990–2021.

To examine the impact of population aging, population growth, and epidemiologic changes on the global schizophrenia ASIR and ASPR between 1990 and 2021, we conducted a quantitative decomposition analysis (**Figure 9**). The results show that Population is the main contributor to this increase in the global number of schizophrenia cases between 1990 and 2021, followed by the impact of epidemiology. The worldwide growth of schizophrenia prevalence. Population is the primary contributor to this increase, followed by the effects of ageing.

### 3.7 APC modeling analysis

**Figure 10:**
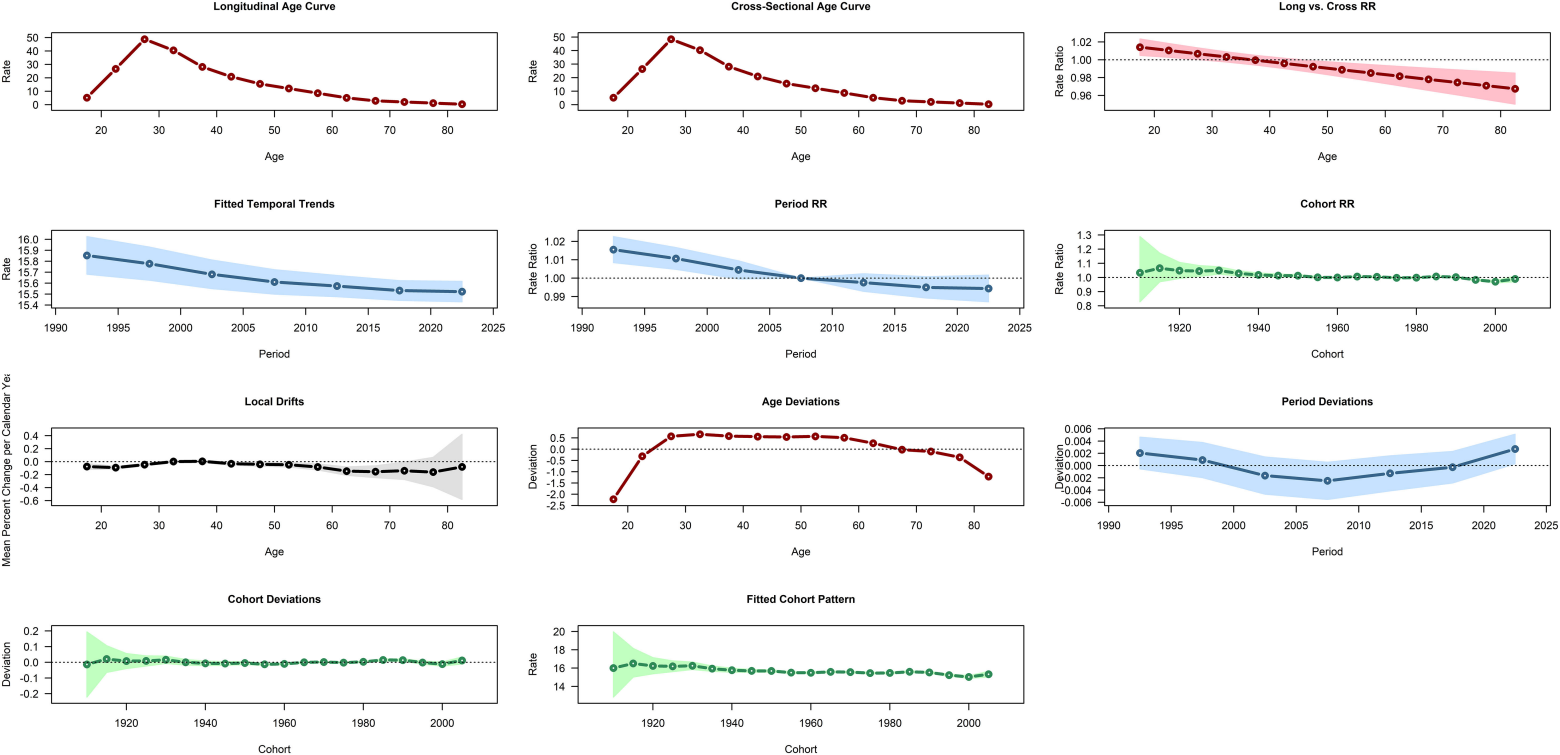
APC analysis of global schizophrenia incidence from 1990 to 2021.

**Figure 10** dissects the driving patterns of global schizophrenia incidence via the Age-Period-Cohort (APC) model. The core finding is that schizophrenia onset clusters in adolescence, peaking at 20–24 years and declining continuously thereafter with increasing age. The period and cohort relative risks (RRs) remain stable over the long term, and the cohort RR shows no significant fluctuations across recent decades (with cohort RRs close to 1.0), indicating minimal differences in incidence risk between recent and earlier birth cohorts. The APC model is analyzed based on two assumptions: the multiplicative effect hypothesis (where incidence is the product of age, period, and cohort effects) and the linear trend hypothesis (positing that the effects of age, period, and cohort change monotonically or linearly along their respective dimensions). Using Locally Weighted Scatterplot Smoothing (LOESS) residuals and permutation tests, a natural breakpoint in the age dimension (rapid increase during 15–24 years, followed by a decline from 25 to 80 years) and a latent breakpoint in the period dimension (around 2000–2005, where the declining rate of the period effect slowed) were identified.

### 3.8 Frontier analysis based on age-standardized DALY

To assess how DALY performs given a country’s or region’s development status, we conducted a frontier analysis based on age-standardized DALY and SDI indices using data from 1990 to 2021. The frontier delineates the age-standardized DALY, which is possible based on the SDI. Overall, countries with higher SDI indices have the lowest effective variance. However, the highest effective variance lies in the middle of the SDI spectrum.

**Figure 11:**
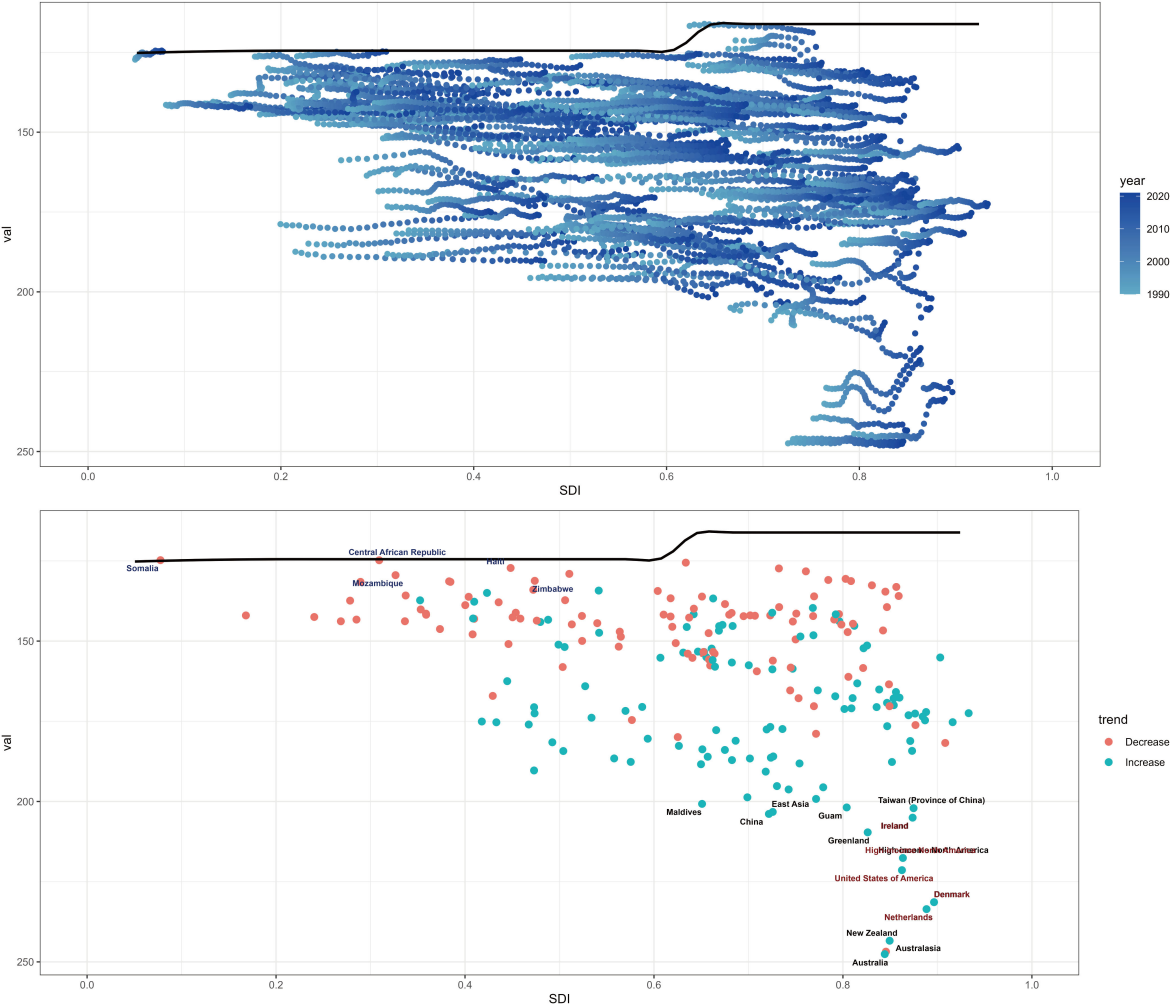
Frontier analysis based on SDI indices and age-standardized schizophrenia from 1990 to 2021.

United States of America, Australasia, Denmark, and Ireland are the countries with the most incredible opportunity to close the gap. It is worth noting that the leading performers are not limited to developed countries-the two countries with low SDI (<0.4), Somalia, Mozambique, and Central African Republic, have very low effective variance, see **Figure 11**.

### 3.9 Future projections of the global burden of schizophrenia

The ARIMA model, as a combination of autoregressive and moving average models, has been widely applied in predicting disease trends due to its effectiveness. The goodness of fit of the model is assessed via the Ljung-Box test; a p-value greater than 0.05 in the test results indicates that the model fits well. For evaluating prediction accuracy, the root mean square error (RMSE), mean absolute error (MAE), and mean absolute percentage error (MAPE) are employed.

**Figure 12:**
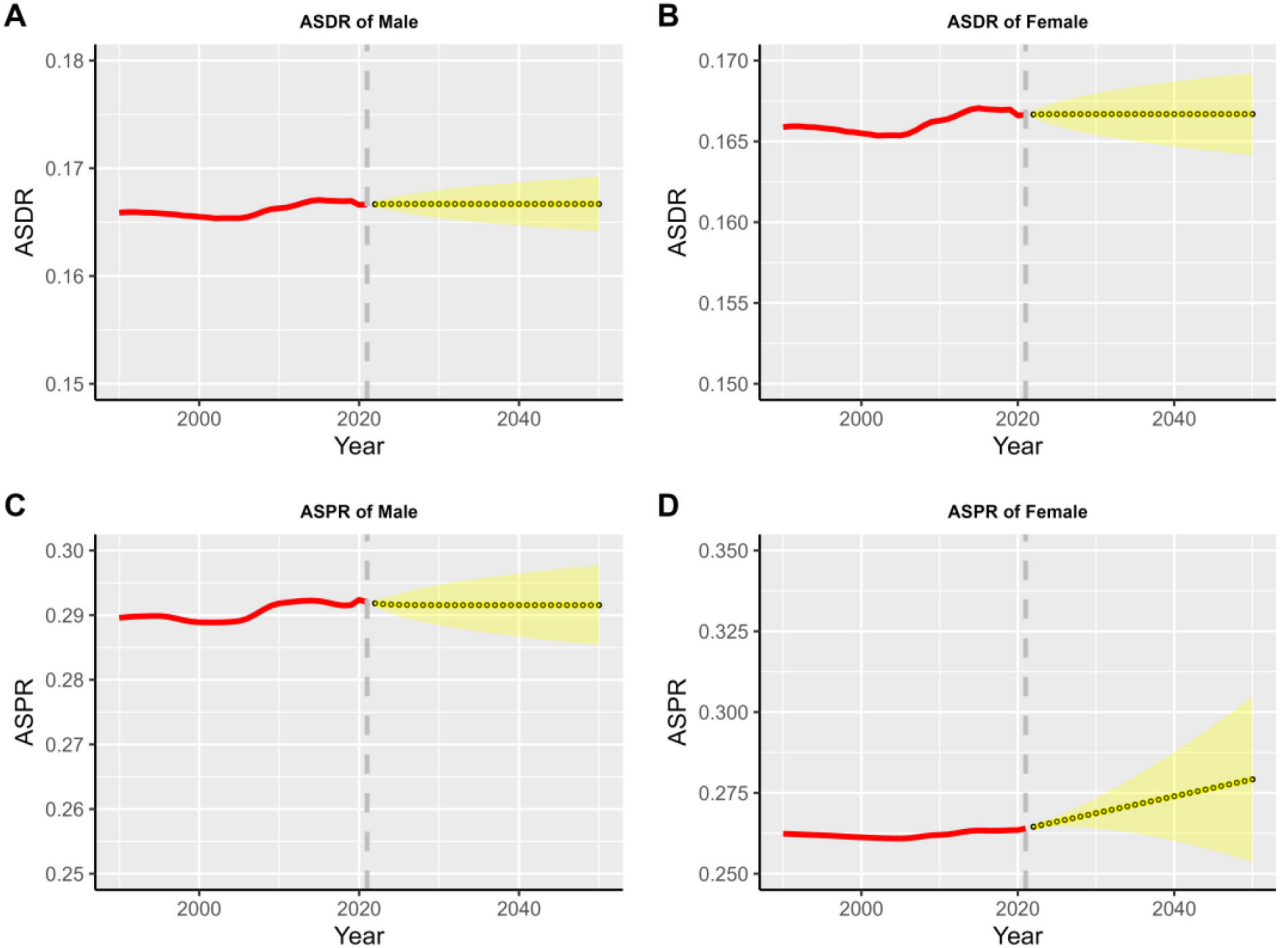
Projected global burden of schizophrenia, 1990–2050.

According to the projections of the ARIMA model (see **Figure 12**), the global burden of schizophrenic illness will continue to grow through 2050. Specifically, the prevalence and incidence rates for both males and females are trending upward and eventually leveling off. This projection highlights the continuing challenge of coping with the disease, with the male population, having higher ASIR and ASPR than females. In addition, the uncertainty in the projection intervals increases progressively over time, reflecting the challenges inherent in long-term disease burden projections.

The long-term projections in this study are based on the extrapolation of historical trends and do not incorporate potential major future variables (such as breakthroughs in mental health policies, socio-economic crises, new public health events, etc.). Therefore, the 95% confidence intervals only reflect statistical uncertainty within the model, rather than all risks in real-world scenarios. Robustness tests indicate that the trends have a certain degree of stability; however, future work should integrate dynamically monitored data (e.g., GBD data updated every 5 years) to revise the prediction parameters.

**Figure 13:**
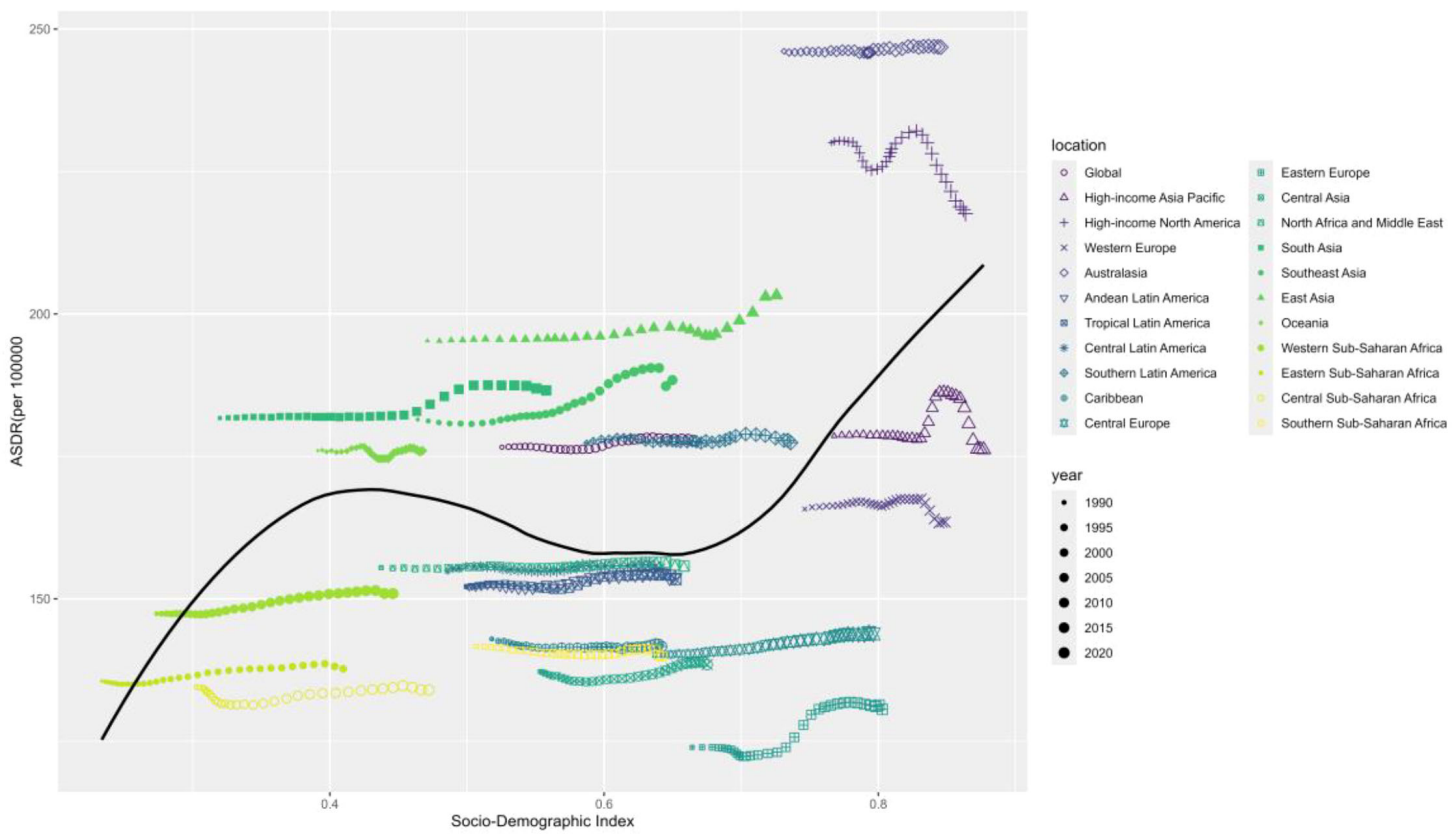
ASDR for schizophrenia in 22 GBD areas, 1990–2021.

Estimates of schizophrenic ASDR are higher than expected for East Asia, Western Europe, South Asia and Australasia based on the SDI from 1990 to 2021. In contrast, Central Sub-Saharan Africa, Eastern Europe, Central Europe, and High-income Asia Pacific had lower than expected levels based on the SDI in all years, see **Figure 13**.

**Figure 14:**
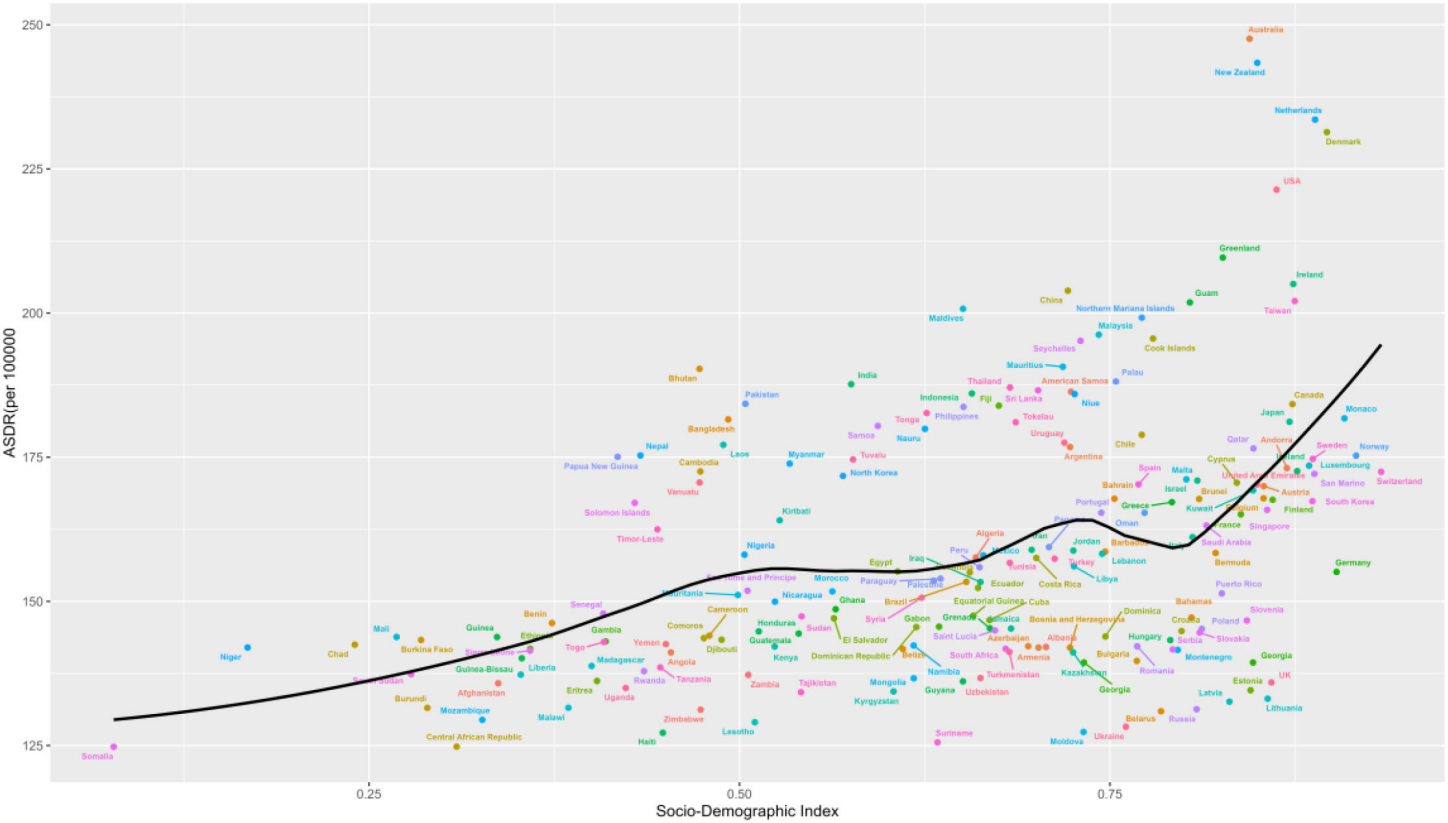
ASDR for schizophrenia in 204 countries and territories in 2021.

Country-level analyses show that the high burden of schizophrenia is not limited to high SDI countries such as the USA, Australia, New Zealand, Denmark, and the Netherlands. The burden of disease is also high in countries and regions with lower SDI, such as Niger, Mali, Chad, Benin, and Burkina Faso, see **Figure 14**.

## 4 Discussion

Schizophrenia is a mental disorder that typically onsets in adulthood, affecting approximately 0.3% of the population (13), the chronic nature of the illness coupled with debilitating symptoms results in a significant impact of the disease on the burden of society. Previous studies have shown that lower socioeconomic levels lead to a higher prevalence of schizophrenia (14, 15). At the same time, the odds of being diagnosed with schizophrenia are much higher among immigrant groups, especially refugees, further emphasizing the role of socioeconomic status in the disease (16).

The rapid advancement of urbanization has increasingly drawn attention to its adverse impacts on human health—particularly mental health. What’s more, the degree of urbanization is a key factor behind the differences in the risk of schizophrenia between urban and rural populations: generally speaking, the higher the level of urbanization and the longer one has lived in an urban area, the higher the risk of developing schizophrenia (17).

Furthermore, certain patients may develop resistance to standard treatment modalities due to the worsening of their condition and more frequent relapses (18). Additionally, substance abuse, including alcoholism and smoking, is prevalent among individuals with schizophrenia, which can lead to comorbid conditions such as malnutrition, diabetes mellitus, vascular complications, blood-borne infections, and Chronic Obstructive Pulmonary Disease (COPD), ultimately resulting in increased disability and mortality (19).

The clinical signs and symptoms of schizophrenia are diverse and vary widely in severity. Schizophrenia requires lifelong treatment, and people with schizophrenia are also at higher risk of suicide attempts and assaults, which can be a daunting task for both patients and their families (20, 21).

Furthermore, we found that the burden of schizophrenia in men remains higher than that in women (22). Men tend to have an earlier onset and more severe symptoms, such as those leading to reduced fertility (23). This is consistent with previous research findings, which attribute these differences to the combined effects of multiple factors including genetics, neurodevelopment, and hormones (24).

The incidence of schizophrenia is most pronounced within the 15–39 age demographic, exhibiting a peak within the 20–54 age range, followed by a gradual decline as age increases. Specifically, the highest incidence is observed in the 20–24 age group, with prevalence reaching its zenith in the 35–39 age cohort before subsequently decreasing with advancing age. Notably, prevalence, incidence, and years lived with disability (YLD) metrics are consistently higher in males across nearly all age categories.

Furthermore, the prevalence of schizophrenia is particularly elevated in the 30–34 age group, a developmental stage that may be characterized by considerable stress. Consequently, we propose that several factors may influence this phenomenon. One such factor is employment status, including career progression or unemployment aspects, which are intrinsically linked to financial stability (25). Familial issues, such as divorce or separation, may also contribute to this increased prevalence (26).

The age range associated with an increased prevalence of schizophrenia is broader in countries with a high SDI compared to those with a low SDI. This observation aligns with previous analyses indicating that the burden of schizophrenia disproportionately affects more affluent urban populations. Such populations may be more vulnerable to particular environmental factors, including migration and urbanization, both of which have been linked to an elevated risk of developing psychiatric disorders, including schizophrenia (27). The comparatively lower prevalence of schizophrenia in low SDI countries may be significantly biased and potentially underestimated. Conversely, the heightened prevalence of schizophrenia in economically stable, high-income countries within high SDI regions may be indicative of greater awareness and reporting of psychiatric disorders, facilitated by superior healthcare infrastructure. Additionally, the high levels of urbanization and dense housing characteristic of high-income countries may contribute to increased rates of schizophrenia, potentially due to elevated stress and pollution levels in these environments (28, 29).

HDI and SDI are proxy indicators for comprehensive socio-economic development. Their association with disease burden may be influenced by complex mediating factors (such as access to healthcare resources, educational level, and poverty level) and may involve bidirectional associations (e.g., increased disease burden may also constrain socio-economic development). Additionally, the association between HDI/SDI and disease burden may be confounded by unobserved variables (such as cultural practices, public health policies, and emergent epidemics). For instance, regions with high HDI may simultaneously have better mental health services and lower poverty rates, with the latter potentially being the direct cause of reduced disease burden.

Numerous healthcare systems within the MENA region have not fully achieved their potential nor delivered an adequate standard of care, primarily due to factors such as economic crises, rapid population growth, a shortage of healthcare professionals, insufficient coverage, political instability, and prevailing stigmas associated with mental illness among the general populace (30). Consequently, these challenges frequently lead to instances of mismanagement, misdiagnosis, or undiagnosed conditions.

The data from this study highlight the need for screening and intervention for schizophrenia before the peak age of its onset. Profound economic, social, and environmental changes will further exacerbate the global disease burden of schizophrenia. Therefore, greater access to healthcare and social support is required to prevent treatment-resistant conditions and worse outcomes, such as suicide, drug overdose, or domestic violence (31).

By analyzing Trends in the burden of childhood schizophrenia, globally and in the five SDI regions, 1990–2021 (S3), we observed that in high-middle SDI regions, around the year 2000, the ASDR, ASIR, and ASPR exhibited peak values and fluctuations. This was attributed to social stressors derived from accelerated urbanization, the detection of previously undiagnosed cases driven by the popularization of diagnostic criteria, and the subsequent establishment of early intervention systems in child psychiatry. Meanwhile, in low SDI regions, although the baseline levels of these three indicators consistently remained the lowest, the disease burden showed a continuous upward trend, resulting from the dual effects of the long-term accumulation of environmental risks such as poverty and the improved diagnostic recognition capabilities promoted by global health initiatives.

While GBD has continued to improve in terms of data and methods for assessing the burden of schizophrenia, it is still necessary to acknowledge some challenges. However, several challenges must be acknowledged. Firstly, due to differences in healthcare infrastructure, resource availability, and clinical practices, variations exist across regions in terms of diagnostic criteria, screening coverage, detection technologies, and reporting completeness. These discrepancies may give rise to apparent differences in disease burden, cannot reflect the true epidemiological changes in incidence rates. Secondly, the task of quantifying and mitigating all variations in prevalence estimates attributable to measurement error presents considerable difficulties. Although the Institute for Health Metrics and Evaluation (IHME) has enhanced its methodologies to address known biases, such as those related to case definitions and survey techniques, the availability of data points necessary for making these adjustments remains limited. Furthermore, there is a notable scarcity of research focused on the risk factors associated with schizophrenia, which could serve as predictive covariates in epidemiological models (32).

The study findings reveal that the age of onset of schizophrenia is slightly earlier in both males and females, with the incidence rising significantly in the 10–14 age group and peaking in the 20–24 age group. This period coincides with a critical stage for educational and professional development, making it crucial to explore potential health outcomes associated with schizophrenia. Individuals with schizophrenia exhibit poor health status, including low socioeconomic status, lack of access to comprehensive health services, and poor health behaviors.

To alleviate the burden associated with schizophrenia, it is imperative to formulate a strategic plan aimed at addressing the escalating socioeconomic disparities. Furthermore, immediate action is required to mitigate the stigma surrounding schizophrenia and to foster a more inclusive and supportive socioeconomic environment for affected individuals.

The long-term projections in this study are based on the extrapolation of historical trends and do not incorporate potential major future variables (such as breakthroughs in mental health policies, socio-economic crises, new public health events, etc.). Therefore, the 95% confidence intervals only reflect statistical uncertainty within the model, rather than all risks in real-world scenarios. Robustness tests indicate that the trends have a certain degree of stability; however, future work should integrate dynamically monitored data (e.g., GBD data updated every 5 years) to revise the prediction parameters.

## 5 Conclusion

Schizophrenia continues to pose a significant public health challenge, and as healthcare systems enhance their screening and identification processes, the already low prevalence of this disorder may experience a substantial increase in the future. Regions and countries with high SDI should prioritize addressing the recent and anticipated rise in schizophrenia risk among birth cohorts. Conversely, nations and areas with low SDI must implement strategies to prevent population growth from contributing to an escalation in schizophrenia prevalence.

By mitigating the prevalence of this mental health condition, the associated burden of comorbidities and related issues can also be reduced, thereby making a meaningful contribution to the overall health of communities and nations. Consequently, initiatives aimed at increasing awareness of schizophrenia among patients, their families, and their social networks, as well as efforts to diminish the stigma surrounding mental illness, could play a crucial role in alleviating the disability burden linked to this disorder.

## Data Availability

The original contributions presented in the study are included in the article/**Supplementary Material**. Further inquiries can be directed to the corresponding author.
