# Supplementary figures and images for: Results of the Global Burden of Disease study for schizophrenia: trends from 1990 to 2021 and projections to 2050
Source: Front Psychiatry. 2025 Sep 5;16:1629032. doi: 10.3389/fpsyt.2025.1629032 (PMC12447577; doi:10.3389/fpsyt.2025.1629032)

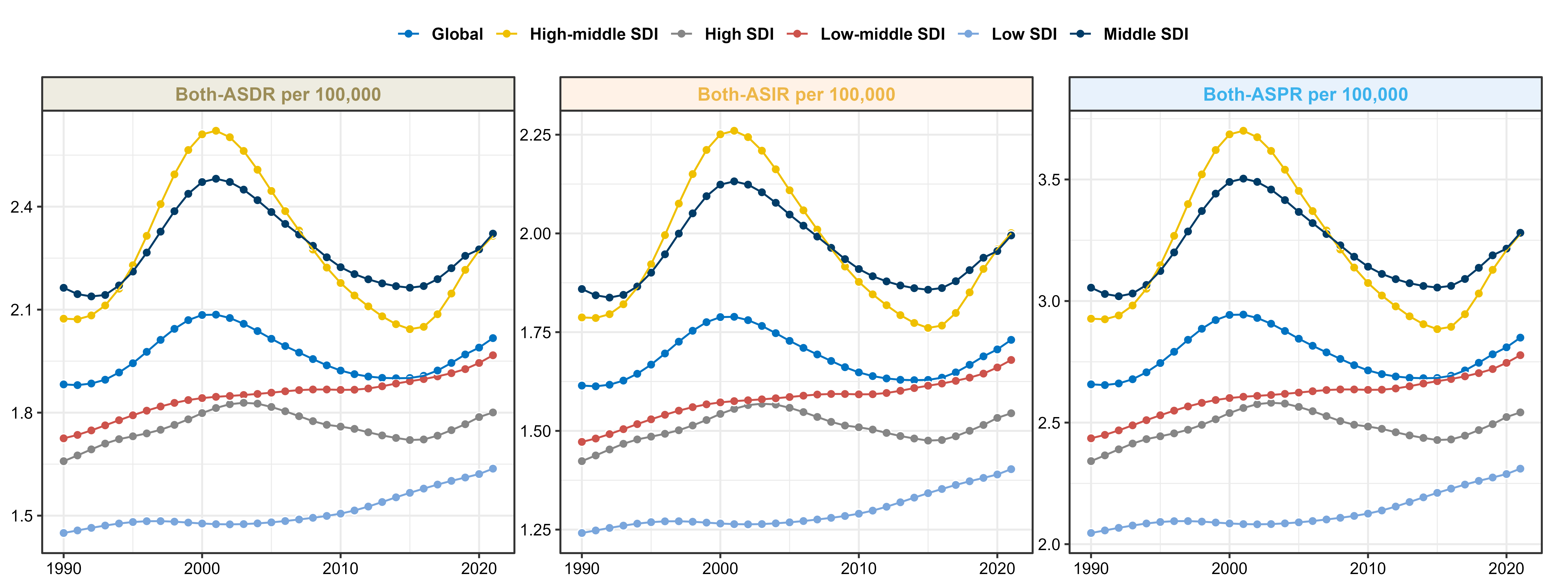

Supplement: Supplementary file 1 [file Image1.png]
